# Magnetic Resonance-guided Inter-fraction Monitoring Opens Doors to Delivering Safer Reirradiation: An Illustrative Case Report and Discussion

**DOI:** 10.7759/cureus.2479

**Published:** 2018-04-14

**Authors:** Rebecca Levin-Epstein, Minsong Cao, Percy Lee, Michael L. Steinberg, James Lamb, Ann C Raldow

**Affiliations:** 1 Department of Radiation Oncology, University of California, Los Angeles, Los Angeles, USA

**Keywords:** mr guided radiotherapy, mri guidance, stereotactic body radiotherapy (sbrt), reirradiation, pelvic recurrences, rectal cancer, bowel toxicity

## Abstract

Locoregional recurrence in the pelvis after definitive treatment for rectal cancer can lead to significant morbidity. Furthermore, the toxicity associated with reirradiation may also negatively impact the quality of life and even survival. Here we present the case of a 39-year-old male with locoregionally recurrent rectal cancer in a left pelvic sidewall lymph node, treated with stereotactic magnetic resonance (MR)-guided ablative radiotherapy after previously receiving long-course chemoradiation that had already exceeded ideal bowel dose constraints. We discuss the distinct advantages of MR-guidance in the setting of pelvic reirradiation, particularly with regard to inter- and intra-fraction visualization of the target and neighboring bowel anatomy. In this context, MR-guidance may allow radiation oncologists to increase target precision and accuracy, while simultaneously decreasing toxicity to neighboring tissues.

## Introduction

Reirradiation to the pelvis presents a clinical challenge to the radiation oncology team. After patients have received definitive radiation therapy for pelvic malignancies, the surrounding tissues have frequently been exposed to doses at or near the maximum acceptable dose constraints [1,2]. When patients require re-treatment in the pelvis, it is important that physicians feel confident in their ability to deliver this treatment safely. Furthermore, when reirradiation is delivered with curative intent, both acute and long-term effects of reirradiation must be considered. Critical structures in the pelvis such as small and large bowel, as well as soft tissue treatment targets, are difficult to image on standard cone beam computed tomography (CBCT), and their position may vary between treatments [3]. Thus, a target may not be reliably visualized during daily image-guided setup, which can result in a geographical miss and increased toxicity to neighboring bowel [3]. Magnetic resonance (MR)-guided radiation therapy (MRgRT) provides a solution to both of these problems, with excellent visualization of bowel as well as non-bony targets [3]. Here, we present a case of MR-guided stereotactic body radiation therapy (SBRT) to a left pelvic sidewall lymph node only six months after completion of long-course chemoradiation for rectal cancer. This case illustrates the value and utility of MRgRT in the setting of reirradiation to the pelvis.

## Case presentation

Our patient is a 39-year-old male who presented with metastatic stage IVa cT2cN2M1a rectal adenocarcinoma located 12 cm from the anal verge with metastases to the liver. He underwent neoadjuvant chemotherapy with seven cycles of capecitabine-oxaliplatin, followed by long-course chemoradiation. This course consisted of intensity modulated radiation therapy (IMRT) at an outside center, 45 Gy in 25 fractions to the pelvis with a subsequent pelvic cone down of 5.4 Gy in three fractions and an additional 3.6 Gy in two fractions to the gross rectal tumor, given concurrently with capecitabine and trastuzumab. He subsequently underwent laparoscopic-assisted low anterior resection with colorectal pelvic anastomosis and diverting loop ileostomy. Pathology from this revealed ypT4N1b rectal adenocarcinoma with lymphovascular invasion. Throughout this period, his liver lesions were successfully treated with several courses of microwave ablation and chemoembolization. Restaging positron emission tomography-computed tomography (PET-CT) scan five months later demonstrated a new single site of FDG-avidity within a 1.1 cm left pelvic sidewall lymph node with no other sites of disease. He was referred for curative intent radiation therapy to this lymph node to treat his only active site of disease.

This patient had already exceeded the ideal dose constraints for bowel tolerance utilized by our institution and as outlined in RTOG 0822 and RTOG 0529 [1,2]. Specifically, his prior radiation treatment included a maximum point dose to the small bowel of 54 Gy, with V (50 Gy) of 16 cc, V (45 Gy) of 105 cc, V (40 Gy) of 198 cc, and V (35 Gy) of 329 cc; the latter two volumes are nearly double the ideal volume receiving 35 and 40 Gy. Large bowel had also exceeded ideal constraints, with V (45 Gy) of 41 cc. However, in the context of this young patient with oligorecurrent disease, we aimed to offer this patient curative-intent treatment.

**Figure 1:**
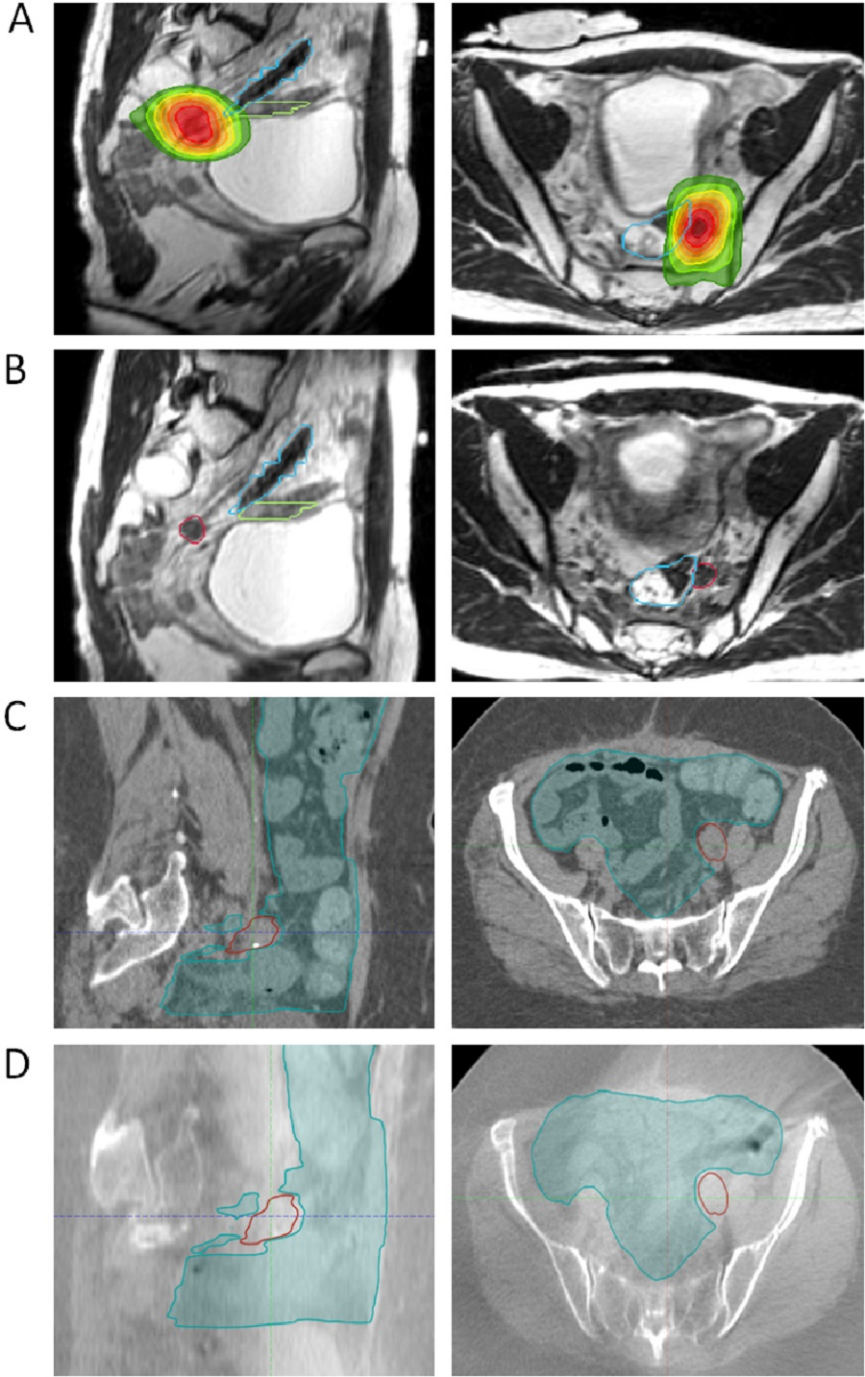
Comparison of simulation scan and daily setup for magnetic resonance (MR)-guided treatment versus computed tomography (CT) onboard imaging. Panels A and B: MR simulation and daily setup scans demonstrating proximity of target lymph node to critical bowel structures, with daily setup visualization of the bowel with MR. Prescription dose 7 Gy x 5. Blue contour: large bowel. Green contour: small bowel. Red contour: gross tumor volume. (A) MR simulation scans, sagittal and axial, with dose wash to 50% isodose line. (B) Sagittal and axial daily setup MR images, treatment day 3. Panels C and D: Images from a similar case to the patient described in this report, but treated with CT guidance. (C) CT simulation scan, with good bowel visualization, compared to (D) Cone beam CT daily setup image, with limited ability to visualize target or bowel.

The patient was thus treated with MR-guided SBRT, 35 Gy in five fractions delivered every other day to the left pelvic sidewall lymph node plus a 3 mm planning target volume expansion (Figure 1, panel A). Cumulative EQD2 (using α/β = 3) maximum point dose to the small and large bowel was 71.5 and 84.5 Gy, respectively.

**Figure 2:**
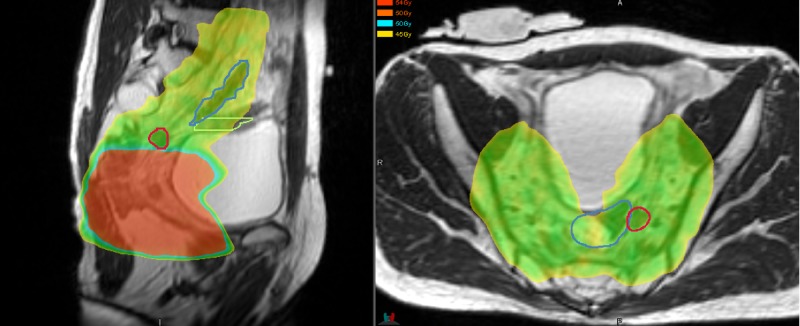
Location of pelvic recurrence in relation to prior radiation field. Red circle = recurrence. Yellow/green = 45 Gy.

The location of the recurrence in relation to the prior irradiated field is demonstrated in Figure 2.

The simulation and daily setup pre-treatment MRI scans were obtained on the ViewRay system (ViewRay, Inc., Oakwood Village, OH) incorporating a 0.35 T MRI. The same imaging protocol using a True Fast Imaging with Steady State Free Precession (TRUFI) sequence was performed to acquire the 3D MRI images with 1.5 mm thickness and a field of view of 50 x 45 x 43 cm without contrast injection. The total acquisition time was 172 seconds.

After image acquisition each day, a physician assessed the target and relation to surrounding anatomy. In the case of movement of critical structures close to the target, the physician had the option to create an adaptive plan that minimized the dose to critical structures. In this case, no adaptive planning was required as the position of the bowel relative to the target did not move significantly. Treatment was delivered using IMRT on the ViewRay system with three Cobalt-60 sources spaced at 120 degrees apart. During treatment delivery, cine MRI imaging at a rate of four frames per second was performed through a sagittal plane to track the intra-fractional motion of the target.

The patient experienced no acute toxicity. One month after completion of SBRT, the patient underwent a successful ileostomy reversal, with excellent progression toward return of normal bowel function. Repeat MRI at two months post-SBRT demonstrated no evidence of disease.

## Discussion

Our case study describes reirradiation with MR-guided SBRT to a left pelvic sidewall lymph node for oligorecurrent rectal adenocarcinoma at a short interval of only six months after long-course chemoradiation to the pelvis. This case was particularly challenging, as ideal bowel dose constraints had already been exceeded from the patient’s prior course of radiation.

The true tolerance of small and large bowel to reirradiation is not well defined. The best data available on bowel toxicity in this context comes from retrospective studies that examine reirradiation for pelvic recurrence after previous long course radiation. Toxicity rates in this setting using 3D conformal radiation and IMRT are quite high, with a limited reirradiation dose achieved (median cumulative dose 77.4–89.4 Gy) [4-6]. Late grade 3-4 toxicity is reported at 21–34%, including GI or GU stricture, obstruction, fistula formation, and long-term requirement for parenteral nutrition [4,6]. Acutely during reirradiation, up to 22% of patients require a treatment break due to acute toxicity [4]. The addition of surgery has also been shown to be an independent predictor for the higher likelihood of experiencing grade 3-4 toxicity [4]. Outcomes from SBRT for pelvic recurrences, as used for our patient, have also been reported using CT-guided systems [7,8]. In a report of SBRT with median 25 Gy delivered to pelvic recurrences (predominantly pre-sacral or pelvic sidewall), 16.7% of patients experienced grade 3 or higher toxicity, including a small bowel perforation requiring surgery and ureteral fibrosis causing hydronephrosis requiring stent placement [7]. Acute toxicity is reported at 39% with higher SBRT doses of median 39 Gy [8].

Locoregional recurrence after long-course radiation therapy followed by surgery is quite common, and is reported at 7.1% at 10 years [9]. With an estimated annual incidence of 38,312 new diagnoses of rectal cancer [10], we can estimate that for each year, 2,720 of these newly diagnosed patients will ultimately experience locoregional recurrence. This also means that an equal number of patients will be at risk for developing the relatively high rate of acute and late severe toxicities described above.

The use of MR guidance for reirradiation to the pelvis is a broad and impactful application for MRgRT. We hypothesize that the techniques described in our case highlight an opportunity to improve the safety of reirradiation, particularly in the SBRT setting with smaller targets. With regard to daily setup, MR-guidance provided the ability to accurately visualize and align to the target, regardless of variations in anatomy such as bowel and bladder distension. On CBCT daily setup, bowel is very difficult to visualize, as are non-bony targets (Figure 1), making the treatment more prone to errors such as geographical misses and delivery of high doses to bowel. An additional significant advantage of MRgRT in this case was the opportunity for daily adaptive planning on an as-needed basis to avoid critical structures, particularly bowel that may migrate into the field as part of normal day-to-day variation. In this patient’s treatment, no adaptive plans were required.

During treatment delivery, MRgRT provided additional benefit. With intra-fractional gating, treatment delivery pauses if the target has moved outside of a pre-defined tracking boundary, i.e., the 3 mm expansion from gross tumor volume (GTV). Thus, MRgRT ensures that if bowel has migrated into the treatment field, pushing the lymph node away from its original starting point from the daily setup, treatment will pause rather than delivering treatment-level dose to that traveling segment of bowel.

MRgRT not only has the potential to improve patient toxicity, but also to improve physician confidence in re-treating this difficult area, and as a result, may increase the number of patients who go on to receive potentially curative pelvic reirradiation.

## Conclusions

By using daily MR guidance to visualize the target and critical structures, MRgRT allowed us to minimize dose to previously irradiated neighboring bowel and to deliver a curative-intent dose to the single remaining site of active disease in a young patient with oligorecurrent rectal cancer. MRgRT was especially useful in this case, in which bowel had already received dose levels above normally accepted constraints. With MRgRT we felt confident in our ability to deliver precise, on-target treatment, with minimized toxicity because of inter- and intra-fraction visualization of anatomy. These advantages are applicable to all histologies with pelvic recurrence, offering the advantages of MRgRT in the locally recurrent setting to a much broader population of cancer patients.
